# Reasons for living and dying in suicide attempters: a two-year prospective study

**DOI:** 10.1186/s12888-018-1814-8

**Published:** 2018-07-20

**Authors:** Juliane Brüdern, Annabarbara Stähli, Anja Gysin-Maillart, Konrad Michel, Thomas Reisch, David A. Jobes, Jeannette Brodbeck

**Affiliations:** 1 Hospital of Psychiatry Muensingen, Hunzigenallee 1, CH-3110, Muensingen, Switzerland; 2 University of Bern, Gesellschaftsstrasse 49, CH-3012 Bern, Switzerland; 3 Translational Research Centre, University Hospital of Psychiatry, University of Bern, Murtenstrasse 21, CH-3008 Bern, Switzerland; 4 University Hospital of Psychiatry, Murtenstrasse 21, CH-3008 Bern, Switzerland; 5 Translational Research Centre, University Hospital of Psychiatry, University of Bern, Hospital of Psychiatry Muensingen, Hunzigenallee 1, CH-3110, Muensingen, Switzerland; 6 The Catholic University of America, 314 O’Boyle Hall, N.E., Washington DC, 20064 USA; 7 University of Bern, Fabrikstrasse 8, CH-3012 Bern, Switzerland

**Keywords:** Suicide ideation, Suicide attempt, Depression, Protective and risk factors

## Abstract

**Background:**

The internal suicide debate hypothesis assumes that in a suicidal crisis, individuals are involved in an internal struggle over whether to live or die. Reasons for living (RFL) and Reasons for dying (RFD) are important individual reasons for staying alive (e.g. family) or wanting to die (e.g. hopelessness) and reflect this internal motivational conflict of the suicidal mind. The aim of this study was to explore the association between RFL and RFD of suicide attempters and current and future suicide ideation and behavior.

**Method:**

The sample consisted of 60 patients who were admitted at a psychiatric emergency unit in Switzerland following an attempted suicide. They received treatment as usual, participated in an assessment interview and completed self-report questionnaires. Additionally, they were instructed to write down up to five individual RFL and RFD. The number of RFL and RFD responses, depressive symptoms, and suicide ideation were assessed at baseline and 6, 12, and 24 months follow-up. Outcome measures were suicide ideation and repeated suicide attempts. Multiple imputations were used in order to address missing data.

**Results:**

The number of RFD responses was the strongest predictor for increased suicide ideation at baseline. The number of RFL responses was not associated with suicide ideation and reattempts. RFD, depressive symptoms, and baseline suicide ideation predicted subsequent suicide reattempt up to 12 months later in simple regression analyses. Mediation analyses suggested that RFD mediated the effect of depressive symptoms at baseline on suicide ideation at 12-months follow-up.

**Conclusion:**

RFL were unrelated to the mental health of study participants and did not function as protective factor against suicide risk. RFD may be an important motivational driver in the suicidal process. Clinical interventions should focus more on the reduction of RFD than on RFL in suicidal individuals.

## Background

Every year, over 800,000 individuals worldwide die by suicide and between 10 and 20 million individuals attempt suicide [1]. In many cases suicidal thoughts are antecedents of a suicide attempt and completed suicide [2]. Among suicide ideators, about one third go on to act on them with a suicide attempt, and 60% of these transitions occur within the first year after onset of suicidal thoughts [3]. Although suicidal behavior is strongly associated with mental disorders, no linear relationship exists; the vast majority of people with mental disorders do not experience suicidal behavior [4]. Thus, psychiatric disorders as risk factors for suicidal behavior have only limited predictive power [5]. Therefore, we need to better understand the motivational processes that lead to suicidal thoughts and why some individuals cross the threshold to act on them. This is particularly important for clinical interventions that address the reduction of suicide ideation as an antecedent to a suicide attempt.

One approach that elaborates that motivational view is the internal suicide debate hypothesis [6]. The hypothesis involves the assumption that suicidal individuals are often entangled in a struggle over whether to live or die and weigh up between reasons for living (RFL) and reasons for dying (RFD). Linehan et al. [7] further examined the life-oriented aspect of this debate and introduced the Reasons for Living Inventory (RFLI). Prospective studies showed that individuals with few reasons for living were at increased risk for developing suicidal thoughts [8] and attempting suicide [9]. A recent study of Cwik et al. [10] strikingly illustrated, that RFL moderated the relationship between depression and suicide ideation. Participants who reported more RFL experienced less suicide ideation even at the highest severity of depression as compared to participants with only a small number of RFL. In line with these results, Gutierrez et al. [11] showed that a low score on the “RFL-Survival and Coping Beliefs”-subscale was associated with an increased suicide risk. However, in this study internal risk factors (e.g. hopelessness and repulsion by life) were more useful in identifying individuals with an increased suicide risk compared to protective factors measured by the RFLI.

Nevertheless, Gutierrez et al. [11] highlight the importance of assessing both ends of the suicidality continuum, reasons for living and dying, in order to get a more detailed picture of suicide risk and to decide about suitable intervention. For example, a person with an increased suicide risk but a strong sense of responsibility to his or her family and fear of suicide might benefit from outpatient therapy. Without these protective factors, hospitalization might be more appropriate. Therefore, covering both facets of the internal ambivalence in suicidal individuals might be essential for obtaining a more comprehensive evaluation of suicide risk. Moreover, interventions addressing risk factors while simultaneously increasing reasons for living should be more effective than those only concentrating on risk factors. For a systematic evaluation of the motivational drivers involved in the suicidal process, more specifically a person’s attraction to life and death, Jobes and Mann [12] developed the “Reasons for living (RFL) and Reasons for dying (RFD) Assessment”, which is part of the Suicide Status Form III [13]. It is a self-report assessment for measuring quantitative and qualitative characteristics of the internal suicide debate that prompts participants to write down up to five individual reasons for staying alive (RFL) vs. wanting to die (RFD) (see Table 1).

**Table 1:** Typical Reasons for living and Reasons for dying

Reasons for living	Reasons for dying
My husband	No more pain
Working in the bookstore	Feeling alone
Music	To stop hurting others
I think things will work out	

Harris et al. [14] investigated the RFL and RFD in 1016 participants classified as high suicidal vs. non-suicidal. Participants with a greater wish to die than to live and a high total-score in the Suicide Behaviors Questionnaire – Revised [15] were categorized as high suicidal and vice versa for the non-suicidal group. They found that the non-suicidal group produced significantly more RFL and fewer RFD than high suicidal respondents. To date, there is no research on the number of RFL and RFD in suicide attempters. The current study intended to close this gap and investigated the quantitative RFL and RFD responses in suicide attempters and their influence on current and future suicidality.

### Aims of the study

1. The first aim of the study was to compare the number of RFL and RFD in individuals with a recent suicide attempt. We assumed that individuals would report significantly more RFD than RFL immediately after a suicide attempt.
2. The second aim was to examine the influence of the number of RFL and RFD at baseline on concurrent suicide ideation at baseline as well as on suicide ideation at 6 months (T2) and 2 years (T4) after the index suicide attempt. Here, we hypothesized that a higher number of RFD would indicate a higher degree of suicide ideation, whereas a higher number of RFL would have a protective effect on current and future suicide ideation.
3. Third, we wanted to investigate whether the number of RFD would mediate the relationship between depression and suicide ideation.
4. Finally, we aimed to explore whether the number of RFL and RFD would have a predictive value on the occurrence of a repeated suicide attempt during a one-year (T1-T3) and a two-year follow-up (T1-T4).

## Methods

### Participants

The sample consists of participants who were admitted at the emergency unit of the Bern University General Hospital following an attempted suicide. The present study, which was part of a larger randomized controlled trial [16], included 60 patients who gave written informed consent and received routine psychiatric treatment (inpatient, day patient, and individual outpatient care). Exclusion criteria were insufficient mastery of the German language, serious cognitive impairment, psychotic disorder, and residency outside the hospital catchment area. Table 2 presents baseline characteristics and clinical diagnoses of the patients. Participants were Caucasians, had an average age of 39 years, and half of them were female. Twenty-five percent were married and 32% had children. Sixty percent were diagnosed with an affective disorder, 47% with a neurotic and stress-related disorder, and 33% a substance abuse disorder. The mean number of diagnoses was 2 (*M* = 2.1, *SD* = .93, range 1–4). Twenty-two percent reported one prior suicide attempt; 35% had a history of multiple (two or more) prior suicide attempts.

**Table 2:** Characteristics of the participants at baseline (*N* = 60)

	*M*	*SD*	*n*	*%*
Age	39.15	14.58		
Male sex			30	50.00
Relationship (yes)			21	35.00
Children (yes)			19	31.66
Employment				
- Unemployed, disability pension, sick leave			26	43.33
- Employed, in training, in education			34	56.66
Previous suicide attempts				
- none			26	43.30
- 1			13	21.70
- 2			10	16.70
- > 2			11	18.30
Diagnoses				
- F1: Mental and behavioural disorders due to psychoactive substance use			20	33.33
- F3: Mood (affective) disorders			36	60.00
- F4: Neurotic, stress-related and somatoform disorders			28	46.66
- F5: Behavioural syndromes associated with physiological disturbances and physical factors			1	1.66
- F6: Disorders of adult personality and behaviour			12	20.00
BDI sum	18.32	12.25		
BSS mean	9.05	9.15		
Number of reasons for living (RFL)	3.45	1.35		
Number of reasons for dying (RFD)	1.90	1.55		

*BDI* Beck Depression Inventory, *BSS* Beck Scale for Suicide ideation

### Measures

#### Socio-demographic questionnaire

The Sociodemographic Questionnaire (DEMO) [17] is a 31-item questionnaire that assesses personal, sociodemographic, and health-related data, including information on suicidal behavior. The DEMO asks for the frequency of suicide ideation and self-harm in the last 6 months as well as for the number of suicide attempts in the last 6 months and during lifetime.

#### Beck depression inventory

The Beck Depression Inventory (BDI) [18] is a 21-item self-report measure to assess the severity of a patient’s current level of depression including affective, cognitive, motivational, behavioral, and somatic components. Items are scored on a 4-point Likert scale (0 = nonexistent to 3 = severe) and are summed up. A score from 18 or above indicates significant depressive symptoms. The German version has demonstrated good validity [19] and for the current study, Cronbach alpha was .88.

#### Beck scale for suicide ideation

The Beck Scale for Suicide Ideation (BSS) [20] is a 21-item self-report instrument and was used to assess the intensity of the patient’s attitudes, behaviors, and plans related to suicidal behavior. The items are scored on a 3-point Likert Scale and are summed up except of the last two items. They assess the number of previous suicide attempts before the index suicide attempt and the strength of the desire to die during the index suicide attempt. The higher the respondent’s total score, the higher is their suicide risk. The German version has demonstrated a very good internal consistency with a Cronbach alpha of .94 [21] and for the current study, Cronbach alpha was .93.

*Reasons for Living and Dying* from the Suicide Status Form (SSF-III) [13]. Participants were instructed to write down up to five RFL and RFD respectively on the SSF-III (see Table 1). For current analyses, we were interested in the numbers of RFL and RFD responses.

### Attrition analysis

All patients took part in a single clinical interview at baseline, which included a structured suicide risk assessment using the “Suicide Status Form” [12]. The median delay time between the suicide attempt and the interview was 17.5 (interquartile range 9.0–34.5) days. They filled out the DEMO) [17], the BDI [18], the BSS [20], and the SSF-III [13] at baseline (T1) and after 6 (T2), 12 (T3), and 24 (T4) months. Six months (T2) after baseline 71.7% of the participants completed the questionnaires; 12 months (T3) after baseline the response rate was 60% of the baseline sample and at the two-year follow-up (T4), 63.3% of the baseline sample took part. Complete data over all included measurement points was obtained by 46.7% of the baseline sample. Data on repeated suicide attempts were primarily assessed with the DEMO [17]. Additionally, we complemented self-reported data by searching medical records and contacting involved health professionals in order to complete data regarding suicidal behavior. Within 12 months after baseline, one patient died by suicide and three died of natural causes.

Overall attrition was significantly associated with a higher number of RFL at baseline (*r* = .26, *p* = .040), stronger psychological pain at the time of the suicide attempt (*r* = .29, *p* = .025), and not being in outpatient treatment between T2 and T3 (*r* = −.33, *p* = .049). Attrition at T2 was associated with fewer suicide attempts (*r* = −.34, *p* = .007), stronger psychological pain at the time of the suicide attempt (*r* = .27, *p* = .033), and fewer outpatient and inpatient treatments before baseline (*r* = − .35, *p* = .008, *r* = −.34, *p* = .007, resp.). Attrition at T3 was associated with stronger psychological pain at the time of the suicide attempt (*r* = .26, *p* = .041) and more psychotherapy sessions during the previous 6 months before the index suicide attempt (*r* = .27, *p* = .040). Attrition at T4 was associated with a higher number of RFL at T1 (*r* = .26, *p* = .048), a lower number of psychotherapy sessions in the 6 months prior to T3 (*r* = .40, *p* = .016). Under the assumption of missing at random, we used multiple imputations for missing data (see data analysis section).

### Statistical analysis

Under the assumption of missing at random, we computed multiple imputations for all variables included in the analyses based on Markov Chain Monte Carlo simulations with the Bayesian estimator in Mplus version 7.4 [22]. A total of 40 imputed datasets were generated and results are reported on pooled analyses of these datasets. Sensitivity analyses yielded similar results using the imputed and the non-imputed datasets. Pearson correlation were used for two continuous variables, tetrachoric correlations for two binary, biserial correlations for one continuous and one binary or ordered polytomous variable, and polychoric correlations for a binary and an ordered polytomous variable. To analyze predictors of the BSS score and repeated suicide attempts, we used robust linear and logistic regression analyses. In the multiple regression analyses, we entered all correlations with a *p*-value <.09 simultaneously.

We conducted cross-sectional path analyses at baseline to investigate whether the number of RFD mediated the relationship between the BDI score and BSS score at baseline. Furthermore, we conducted longitudinal analyses with BDI at baseline, number of RFD at T2, and BSS at T3. This temporal sequence of the variables suggests a directional link from the predictor to the outcome variables via the mediator. It accounts for the implication of the temporal relation with depression occurring before the RFD and the RFD occurring before suicide ideation and thus the notion that the depression at baseline affects the number of RFD 6 months later and the RFD affect the level of suicide ideation at the 12-month follow-up [23].

## Results

### Numbers of reasons for living and reasons for dying and socio-demographic factors

After the index suicide attempt at baseline participants reported significantly more RFL (*M* = 3.45, *SE* = 1.35) than RFD (*M* = 1.90, *SE* = 0.55, *t*(59) = 5.71, *p* < .001), which corresponds to a strong effect (*d* = 1.00). Therefore, our hypothesis that patients immediately after a suicide attempt had more RFD than RFL was rejected. Even though significance level was just not reached, participants with at least one prior suicide attempt produced more RFD (*M* = 2.24, *SE* = 0.28) than participants with no prior suicide attempt (*M* = 1.46, *SE* = 0.25, *t*(58) = − 1.96, *p* = .054, *d* = 0.53). There was no difference regarding RFL between those with and without prior suicide attempt (*M* = 3.38, *SE* = 0.22 vs. *M* = 3.54, *SE* = 0.27, *t*(58) = .44, *p* = .660, *d* = 0.12).

Table 3 presents the zero-order correlations of socio-demographic variables, RFL and RFD at baseline and 6 month (T2) later, BDI at baseline, BSS scores at T1 - T4, and repeated suicide attempts. RFL and RFD responses at baseline did not correlate (*r* = −.05, 95% BCa CI [−.31, .24], *p* = .697). The number of RFL at baseline and at T2 were not significantly related to socio-demographic and clinical sample characteristics or any other variables (all *p* > .05). However, RFD at baseline correlated with BDI at baseline (*r* = .56, 95% CI [.37, .75], *p* < .001) and BSS at baseline (*r* = .70, 95% CI [.54, .86], *p* < .001), BSS at T2 (*r* = .53, 95% CI [.31, .76], *p* < .001), and BSS at T4 (*r* = .29, 95% CI [.04, .53], *p* = .021).

**Table 3:** Correlations between sociodemographic variables, depression and suicide-related variables

	Age	Sex	Part-ner	Child-ren	Prev. SA	RFL T1	RFD T1	RFL T2	RFD T2	BDI T1	BSS T1	BSS T2	BSS T3	BSS T4	Rep. SA 0–12 m
Male Sex ^a^	.13														
Partner ^a^	- .28 ^t^	.06													
Children ^a^	**.49**	- .06	- .29												
Previous SA (0–2)	- .07	- .19	.14	- .27											
RFL T1	.03	.02	.21	- .02	- .13										
RFD T1	.18	.08	- .03	- .16	.25 ^t^	- .05									
RFLT2	- .24	- .11	.15	- .27	.26 ^t^	.23	.19								
RFD T2	- .25 ^t^	- .13	.32 ^t^	**- .41**	.19	- .04	**.51**	**.39**							
BDI T1	- .06	- .25	.08	**- .35**	**.28**	- .19 ^t^	**.56**	- .08	**.48**						
BSS T1	.07	.05	.30 ^t^	**- .45**	**.31**	- .10	**.70**	- .04	**.52**	**.63**					
BSS T2	- .19	- .11	**.45**	**- .37**	.29 ^t^	- .08	**.53**	.23	**.72**	**.55**	**.78**				
BSS T3	- .10	.15	**.37**	- .16	.19	- .05	.14	- .10	**.45**	**.32**	**.37**	**.50**			
BSS T4	.09	- .06	- .05	- .24	**.35**	- .13	**.29**	.02	**.34**	**.43**	**.41**	**.37**	**.50**		
Repeated SA 0–12 months ^a^	.04	- .12	.22	.00	.17	- .18	.34^t^	- .12	.34 ^t^	**.51**	**.48**	**.52**	**.71**	**.54**	
Repeated SA 0–24 months ^a^	- .06	- .19	- .08	- .15	.27	- .05	.23	- .05	.14	**.37**	**.34**	.29	**.36**	**.55**	**.87**

bold = *p* < .05; t *p* < .09; ^a^ 0 = no, 1 = yes

*RFL* Reasons for living, *RFD* Reasons for dying, *BDI* Beck Depression Inventory, *BSS* Beck Scale for Suicide Ideation, *SA* Suicide attempts

### Prediction of suicide ideation at baseline, after six months and after two years

Table 4 presents the results of the simple and multiple regression analyses for predicting the BSS score at baseline, the six-month (T2), and two-year follow-up (T4). Simple regression analyses showed that being in a relationship was significantly associated with a higher BSS score at baseline and BSS at T2. Having children at baseline was significantly related to a lower BSS score at baseline and at T2. RFD at baseline showed the strongest association with BSS score at baseline with an explained variance of 50%. More suicide attempts prior to the baseline, a higher BDI at baseline, and more RFD at baseline consistently predicted a higher BSS score at baseline, at T2, and at T4 with declining effect sizes across time. RFD at baseline still explained 9% of the variance of the BSS score at T4. In contrast, RFL were not associated with suicide ideation.

**Table 4:** Cross-sectional and longitudinal predictors of suicide ideation and repeated suicide attempts

	Simple regressions				Multiple regressions			
	*Std b*	*S.E.*	*p*	*R2*	*Std b*	*S.E.*	*p*	*R2*
BSS score baseline								
Age	.08	.12	.527	.005				
Male sex ^a^	.08	.26	.746	.002				
Partner ^a^	.46	.22	.040	.048	.38	.14	.005	
Children ^a^	−.69	.22	.002	.104	−.33	.14	.017	
Previous SA (0–2)	.31	.13	.018	.074	.05	.08	.534	
RFL t1	−.10	.15	.519	.010				
RFD t1	.70	.06	.000	.493	.52	.09	.000	
BDI t1	.63	.08	.000	.395	.27	.10	.009	.642
BSS score six-month								
Age	−.19	.12	.110	.040				
Male sex ^a^	−.17	.27	.528	.008				
Partner ^a^	.66	.21	.001	.100	.33	.19	.071	
Children ^a^	−.56	.23	.015	.069	.05	.19	.805	
Previous SA (0–2)	.26	.12	.029	.070	.04	.09	.646	
RFL t1	−.08	.14	.568	.008				
RFD t1	.53	.09	.000	.285	−.01	.12	.909	
BDI t1	.55	.10	.000	.308	.12	.13	.343	
BSS t1	.78	.06	.000	.613	.68	.15	.000	.655
BSS score 24-month								
Age	.09	.13	.474	.012				
Male sex ^a^	−.09	.28	.744	.006				
Partner ^a^	−.07	.28	.793	.004				
Children ^a^	−.34	.23	.149	.027				
Previous SA (0–2)	.28	.14	.039	.086	.19	.15	.193	
RFL t1	−.13	.16	.428	.019				
RFD t1	.29	.14	.035	.086	−.08	.19	.664	
BDI t1	.43	.11	.000	.185	.27	.15	.067	
BSS t1	.41	.12	.001	.174	.25	.21	.220	.261
Repeated suicide attempts 0–12 months								
Age	.04	.23	.856	.009				
Male sex ^a^	−.20	.39	.612	.014				
Partner ^a^	.37	.42	.388	.037				
Children ^a^	−.01	.42	.990	.004				
Previous SA (0–2)	.15	.21	.477	.030				
RFL t1	−.18	.19	.331	.036				
RFD t1	.34	.18	.051	.122	−.15	.33	.658	
BDI t1	.51	.19	.006	.264	.35	.25	.156	
BSS t1	.48	.15	.001	.232	.23	.36	.312	.316
Repeated suicide attempts 0–24 months								
Age	−.06	.19	.743	.013				
Male sex ^a^	−.30	.36	.413	.029				
Partner ^a^	−.12	.37	.740	.009				
Children ^a^	−.25	.38	.498	.018				
Previous SA (0–2)	.23	.18	.193	.060				
RFL t1	−.05	.18	.766	.008				
RFD t1	.23	.17	.179	.058				
BDI t1	.37	.18	.034	.143	.25	.23	.267	
BSS t1	.34	.16	.028	.120	.19	.20	.354	.163

^a^0 = no, 1 = yes

*RFL* Reasons for living, *RFD* Reasons for dying, *BSS* Beck Scale for Suicide ideation, *SA* Suicide attempts

As a next step, we computed multiple regression analyses to investigate the unique contribution of all significant predictors in the simple regression analyses controlling for the other predictors. Concurrent associations at baseline confirmed the unique contributions of being in a relationship, having children, a higher number of RFD, and a higher level of depression for predicting suicide ideation. However, previous suicide attempts were no longer significant. Again, RFD at baseline were the strongest predictor; all variables together explained 64% of the variance of suicide ideation at baseline (BSS T1).

We examined longitudinal associations of the predictor variables at baseline on BSS score at T2 controlling for BSS at baseline. Multiple regression analyses showed that only BSS score at baseline remained a significant predictor and had a unique contribution to the BSS at T2. The explained variance was again 65%. Regarding the long-term associations after 2 years (T4), no baseline predictor had a unique contribution to the BSS at T4. The amount of explained variance of the BSS score at T4 declined to 26%.

### Mediation analyses

Table 5 presents the results of the path models testing whether RFD mediated the association between depression (BDI) and suicide ideation (BSS). We found a significant indirect effect (*STD b* = .29, *p* < .0001) of the level of depression on the level of suicide ideation via the number of RFD. The direct relationship between depression and suicide ideation remained significant, but the standardized regression coefficient declined from .63 to .34. Thus, a partial mediation was confirmed. The model explained 57% of the variance of suicide ideation at baseline.

**Table 5:** Results of the mediation analyses

	Std b	S.E.	p	R2
Outcome BSS t1				
Simple regression				
BDI t1 -- > BSS t1	.63	.08	.000	.395
Mediation model including RFD t1				
Direct paths				
BDI t1 -- > BSS t1	.34	.10	.001	
BDI t1 -- > RFD t1	.56	.08	.000	
RFD t1 -- > BSS t1	.51	.09	.000	
Indirect path				
BDI t1 -- > BSS t1 via RFD t1	.29	.06	.000	.572
Outcome BSS t3				
Simple regression				
BDI t1 -- > BSS t3	.32	.14	.022	.107
Mediation model including RFD t2				
Direct paths				
BDI t1 -- > BSS t3	.13	.15	.373	
BDI t1 -- > RFD t2	.48	.12	.000	
RFD t2 -- > BSS t3	.38	.15	.010	
Indirect path				
BDI t1 on BSS t3 via RFD t2	.19	.08	.024	.223

t1 = baseline; t2 = six-month follow-up; t3 = 12-month follow-up

*BDI* Beck Depression Inventory, *BSS* Beck Scale for Suicide Ideation; *RFD* Reasons for dying

As a next step, we repeated these analyses using longitudinal data to test whether RFD at T2 mediated the relationship between the BDI at baseline and BSS at T3. Results confirmed a significant indirect effect (*STD b* = .19, *p* = .024) even though smaller than the cross-sectional indirect effect. The direct effect of depression at baseline on suicide ideation 12 month later disappeared, confirming a full mediation. The model explained 22% of the variance of suicide ideation at 12-month follow-up.

### Prediction of suicide reattempts within one and two years after baseline

Table 4 reports the results of the logistic regression analyses to predict suicide reattempts. Within the 12-month period after baseline, 23.3% (*n* = 10) of the participants reported another suicide attempt. In line with the previous analyses, BSS and BDI at baseline predicted suicide reattempts after 12 months (T1-T3) in the simple regression analyses. The number of RFD at baseline just missed the level of significance (*p* = .051). In contrast to the prediction of suicide ideation, having a partner, having children or previous suicide attempts before baseline did not predict suicide reattempts. Multiple regression analyses explained 32% of the variance in suicide reattempts within 1 year but no single predictor remained significant. Within the two-year period from baseline, 36.7% (*n* = 16) of the participants reported another suicide attempt. BDI and BSS at baseline, but not RFD at baseline predicted significantly suicide reattempts after 2 years (T1 – T4) in the simple regression analyses. The multiple regression model explained 16% of the variance and again no single predictor remained statistically significant.

## Discussion

### Numbers of reasons for living and reasons for dying and their association with socio-demographic and clinical variables

We investigated the number of RFL and RFD responses and their association with baseline and future suicide ideation and suicide reattempts. Against our hypothesis and a previous study where highly suicidal individuals reported more RFD than RFL [14], we found that study participants reported significantly more RFL than RFD after the index suicide attempt. The numbers of RFD and RFL in our sample was comparable to non-suicidal individuals in Harris et al.’s study [14]. The concept of the suicidal mode may explain this finding. The suicidal mode is an out-of-the-ordinary state of mind which has a time-limited nature of activation [24]. When the mode is activated, the person is cognitively and affectively restricted to suicidal thoughts and feelings of hopelessness and helplessness. In most cases the suicide attempt leads to a reduction of inner tension and the deactivation of cognitive restriction—a cathartic effect. Afterwards, individuals often feel relief and again have access to life-oriented goals [25]. It might be that at time of data collection important RFL were again accessible for study participants.

We further found that the number of RFL and RFD responses at baseline were unrelated. This suggests that RFL and RFD represent two different psychological dimensions rather than a continuum. Interestingly, the number of RFL did not correlate with any socio-demographic and clinical sample characteristics. Against our hypothesis, the number of RFL was not associated with the degree of depression at baseline nor suicide ideation and suicide reattempts during the two-year follow-up. Thus, the number of RFL was not a marker for suicide risk. This is not in line with the finding of Cwik et al. [10], where RFL moderated the association between depression and suicide ideation, and contradicts previous research, where fewer RFL were associated with an increased risk of suicidal thoughts and reattempts [8, 9]. A possible explanation for this finding might be the different samples. Some of the previous studies have investigated only suicide ideators, not attempters [9–11]. These might be two different populations. RFL may have a protective effect in ideators, but this effect might disappear in attempters who experienced a strong impulse to act on suicide ideation.

In accordance with our hypothesis, a higher number of RFD was associated with higher levels of depression at baseline as well as a higher degree of suicide ideation at baseline and during the two-year follow-up. Similarly, participants with a history of suicide attempts showed more depression and suicide ideation compared to individuals with no prior attempt and also produced significantly more RFD then the latter. This is in line with results by Gutierrez et al. [11], where internal risk factors were more critical for identifying suicidal individuals than protective factors.

### Predictors for suicide ideation at baseline

Moreover, multiple regression analyses showed that being in a relationship and having children were predictive for suicidal thinking at baseline. While having children was associated with decreased suicide ideation, having a partner was positively associated with suicide ideation and might be a risk factor for having suicidal thoughts. Possibly, study participants with a partner tended to perceive themselves as a burden to their partner, which could be expressed by a higher level of suicidal thoughts. This would be in line with findings of the interpersonal theory of suicide [26], where a person’s perception of being a burden to others was a predictor for suicide ideation. Furthermore, previous suicide attempts were significantly associated with suicide ideation at baseline and follow-up in the simple regressions, but did not become a significant predictor in the multiple regressions. Therefore, these characteristics may indicate a higher risk for suicide ideation when assessed in an interview or questionnaire, which is clinically relevant in terms of identifying individuals at risk for suicidal behaviors. However, they do not seem to uniquely contribute to the development of suicide ideation when including the other predictors and thus are probably not targets for interventions.

In our study, the number of RFD was the strongest predictor for suicide ideation at baseline. This implies that the number of RFD is a strong risk factor for recent suicidal thoughts. Also, depression was a strong predictor for suicide ideation at baseline which is in line with previous results [5]. However, mediation analyses showed that the influence of depression on suicide ideation at baseline was partially explained by the number of RFD. The mediation effect was replicated longitudinally. These results again emphasize the substantial role of RFD on suicidal thinking in the short and long run.

### Associations of reasons for living and dying with suicide ideation, depression and suicide reattempts

At follow-up, only suicide ideation at baseline was a significant predictor for suicide ideation after 6 months and no clinical baseline predictor became significant in order to predict suicidal thoughts after 2 years. These findings indicate that the number of RFD and other clinical predictors at baseline are primarily related to the current suicidal crisis and that their predictive power in regard to future suicidal states can be seen as only limited.

Regarding suicide reattempts, simple regressions showed that the number of RFD almost reached the significance level and depression, as well as suicide ideation at baseline were significant predictors for reattempting suicide within the one-year follow-up period. In contrast, the number of RFL had no influence on suicidal behavior during 12 months after the index suicide attempt. This again underpins the substantial role of RFD as a motivational risk factor of suicidal behavior. In the multiple regressions, RFD, BDI, and BSS explained 32% of the variance in suicide reattempts, but no single predictor remained significant. This indicates that in our study no specific clinical risk factor was predictive for reattempting suicide within 1 year. It was rather the interplay of all three factors increasing the risk of suicidal behavior and, therefore, depression, suicide ideation, and the number of RFD should be assessed in order to evaluate the risk of suicidal behavior after a recent suicide attempt. Within 2 years after the index suicide attempt, clinical baseline predictors were only marginally predictive in regard to a new suicidal crisis indicating that their predictive power is time limited. The results suggest that a reattempt at least 12 month after the index attempt is more influenced by predictors, which are temporally closer to that event. Moreover, research has shown that previous suicide attempts are a reliable and robust risk factor for future suicidal behavior [26, 27]. Surprisingly and against these findings, previous suicide attempts were not predictive for a repeated suicide attempt in our study. In our sample, 43% of the participants had not experienced a past suicide attempt, thus, it can be argued that there was not enough variability to assess the prediction.

### Limitations and future directions

There are a several limitations that should be taken into account. Data on repeated suicide attempts were primarily assessed with self-report questionnaires. This might be related to issues with under- and over-reporting of suicidal behavior, and a divergence between self-reports and hospital-based data [28]. We addressed this issue by complementing self-reported data with searching medical records and contacting involved health professionals. Moreover, median delay time between the index suicide attempt and assessment at baseline was 17.5 days. This was for example due to surgery or medical interventions after the suicide attempt or longer reflection of patients regarding study participations. This time interval might have led to a recall bias, which might have resulted in an overrepresentation of RFL and an underrepresentation of RFD. Furthermore, participants were instructed to write down a maximum of five RFD and RFL, which might have result to a ceiling effect. The recent study was not able to answer the question whether specific reasons were more or less related to an increased suicide risk. Further analyses will explore this notion and will be reported elsewhere. Finally, the relatively small sample size decreases the statistical power and increases the likelihood of Type II errors. For that reason we decided not to carry out a Bonferroni correction resulting in a further loss of power. Nevertheless, multiple hypothesis testing without a statistical correction can increase the risk of Type I error. Therefore, the results of this study can only be considered as preliminary and replications in larger samples are needed.

## Conclusion

Several conclusions for research and clinical practice can be drawn from these findings. First, we found no strong evidence for the assumption that the RFL and RFD are two poles of a suicidality continuum. They rather represented two different dimensions, which were differentially related with suicidality. In this sample, the number of RFL did not correlate with suicide risk and, therefore, was not confirmed as a protective factor against suicide ideation or suicide reattempts. Therefore, the common clinical assumption that a higher number of RFL is linked with a reduced suicide risk should be considered with caution.

Second, findings of this study suggest that the number of RFD can be considered as a motivational driver of the suicidal process and individuals with a high number of RFD are very prone for a suicidal crisis. In regard of suicide risk management and treatment, individual reasons of a person, which serve as motives to end their life or for a recent suicide attempt, should be carefully assessed and treated. Psychological interventions in suicidal crisis should give priority to the reduction or foster a cognitive defusion from RFD, which could serve as motivational drivers in the suicidal process than the elaborating RFL.

## Abbreviations

ASSIP: Attempted Suicide Short Intervention Program
BDI: Beck Depression Inventory
BSS: Beck Scale for Suicide Ideation
DEMO: The sociodemographic questionnaire
RCT: Randomized controlled trail
RFD: Reasons for dying
RFL: Reasons for living
RFLI: Reasons for Living Inventory
SA: suicide attempts
SSF-III: Suicide Status Form-III
